# Complete Resection of a Massive Synovial Osteochondromatosis of the Hip Using an Anterior Approach: A Report of Two Cases

**DOI:** 10.1155/2017/9806863

**Published:** 2017-09-20

**Authors:** Masanao Kataoka, Koji Goto, Yutaka Kuroda, Toshiyuki Kawai, Ouki Murata, Masayuki Sugimoto, Shuichi Matsuda

**Affiliations:** ^1^Department of Orthopaedic Surgery, Kyoto University, Kyoto, Japan; ^2^Department of Orthopaedic Surgery, Nagahama City Hospital, Nagahama, Japan

## Abstract

Arthroscopic excision of synovial osteochondromatosis of the hip is commonly performed. However, when the lesion extends to the extra-articular space of the hip joint, excision using arthroscopy becomes difficult. Although surgical dislocation of the hip with a trochanteric flip osteotomy is commonly used, manual access to the inferomedial portion of the acetabulum remains difficult. In this case report, we describe arthroscopic resection followed by open surgery using an anterior approach with or without surgical dislocation to excise a synovial osteochondromatosis of the hip that had extended to the extra-articular space and formed a herniation sac. Excision was completed without complications. An anterior approach with or without surgical dislocation should be considered as a surgical option for the treatment of a massive synovial osteochondromatosis of the hip joint.

## 1. Introduction

Although a synovial osteochondromatosis can occur in any joint, the occurrence of such a lesion in the hip joint is relatively rare. In their report of 53 cases of synovial osteochondromatoses, Maurice et al. [1] found only two that involved the hip. Treatment of a synovial osteochondromatosis includes excision of any intra-articular loose bodies and synovectomy performed using either arthroscopy or open surgery. Complete excision using arthroscopy, however, is difficult and the recurrence rate is high, ranging from 7.1% to 16.7% [2, 3]. We describe the complete excision of a massive synovial osteochondromatosis that had extended to the extra-articular space to form a herniation sac in the inferomedial region of the hip joint. Although surgical dislocation with a trochanteric flip osteotomy is commonly used to excise a synovial chondromatosis of the hip [4, 5], we selected arthroscopic resection followed by open surgery using an anterior approach [6] to secure access to the herniation sac with or without surgical dislocation.

## 2. Case Presentation

Case 1 was a 65-year-old woman who reported continuous left hip pain and limited range of motion. She was referred to our hospital in June 2013. During the first consultation, physical examination revealed limited range of motion of her left hip due to pain. Her Merle d'Aubigné-Postel score was 9 points, with the following distribution: pain, 2 points; walking ability, 3 points; and mobility, 4 points.

**Figure 1:**
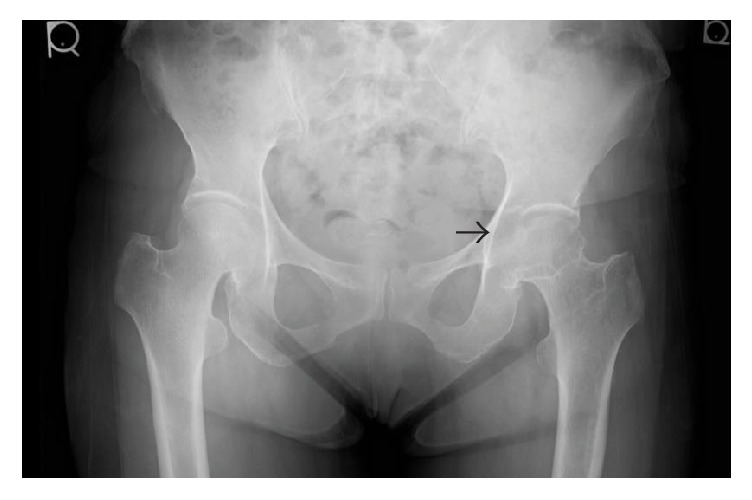
Anterior-posterior radiograph of the hip. Enlargement of the acetabular fossa is shown (black arrow).

**Figure 2:**
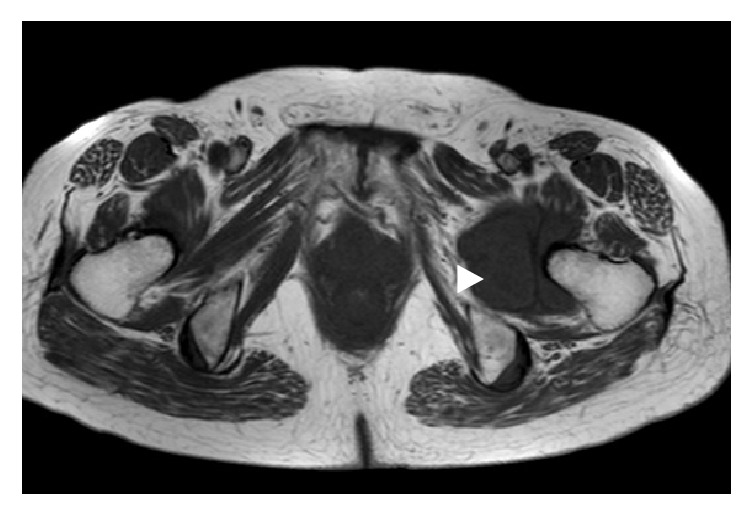
T1-weighted axial magnetic resonance image of the left hip joint. The extra-articular herniation sac is indicated by the arrowhead.

**Figure 3:**
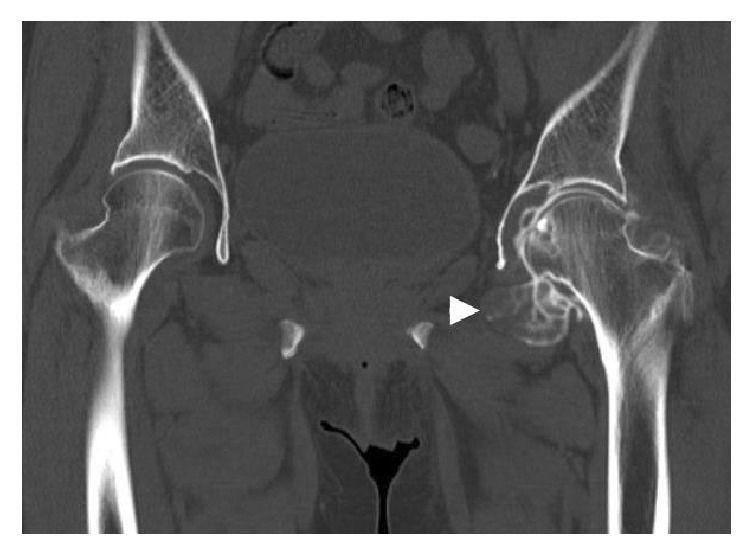
Arthro-computerised tomography scan of the left hip joint. The extra-articular herniation sac is indicated by the arrowhead.

**Figure 4:**
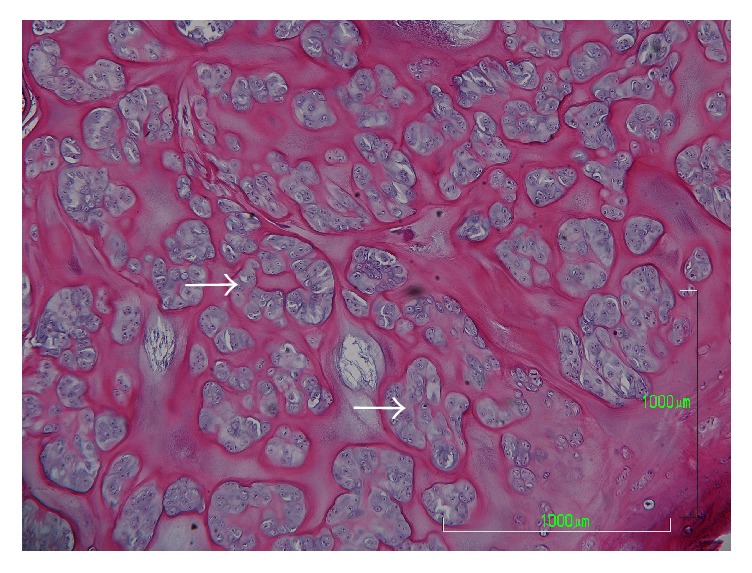
Histological section with haematoxylin and eosin staining. Clustered chondrocytes were observed in hyaline cartilage tissue (arrow).

An anteroposterior plain radiograph of the hip indicated enlargement of the acetabular fossa of the affected hip (Figure 1). Upon magnetic resonance (MR) imaging, coronal and axial images of the left hip joint revealed a large lesion with heterogeneous signal intensity that extended to both the intra-articular and extra-articular joint spaces. A large extra-articular tumour-like lesion appeared to be connected to the inferomedial joint capsule (Figure 2). Arthrography and computed tomography (CT) images of the left hip joint also revealed a honeycomb appearance of the lesion (Figure 3). These examination findings suggested that the tumour was a synovial osteochondromatosis forming a herniation sac. Therefore, our treatment goals were to confirm the diagnosis, extract intra-articular loose bodies, and perform a synovectomy using arthroscopy. Histopathological examination confirmed the diagnosis of a synovial osteochondromatosis (Figure 4). After examining the CT and MR images, we determined that a complete arthroscopic resection would be difficult and that an open anterior approach would be more suitable for manually reaching the herniation sac. Therefore, we performed open surgery using an anterior approach 2 weeks after the initial diagnostic arthroscopy.

**Figure 5:**
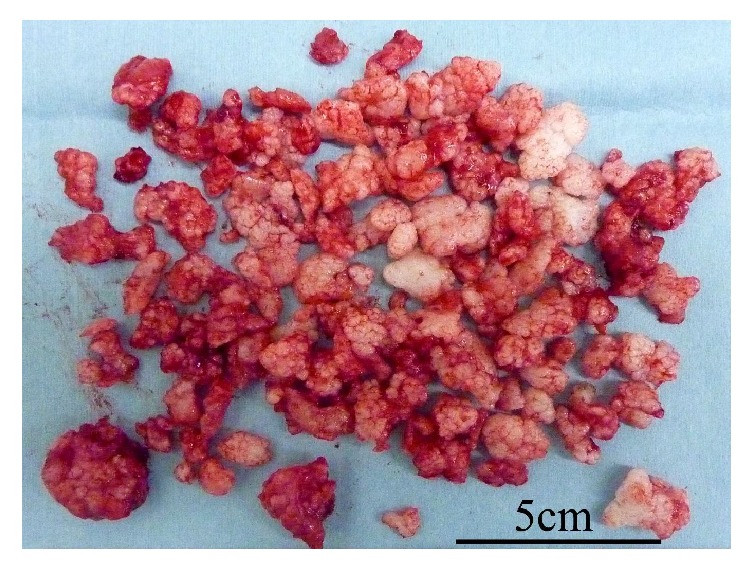
Excised osteochondromas.

The surgical procedure of this case was succinctly reported previously [7], and the salient components of the procedure were as follows. The patient was placed in a supine position, and a 13 cm straight skin incision was made just lateral to the anterior superior iliac spine, extending distally. The lateral femoral cutaneous nerve was identified beneath the fascia and was gently retracted medially. The joint capsule was exposed between the tensor fascia latae and the rectus femoris muscle, and the head of the rectus femoris was transiently detached, with the attachment portion left intact to secure the repair. Near the joint capsule, the ascending branch of the lateral femoral circumflex artery was detected and ligated for capsular exposure. The anterior capsule was opened and the head of the femur was dislocated anteriorly using an external rotation maneuver of the hip. Osteochondromas near the femoral head, including those on the back of the zona orbicularis, were easily removed. Osteochondromas on the acetabular fossa were removed with a curette. Arthroscopic assistance was useful for providing clear visualisation of the acetabular fossa. Osteochondromas in the herniation sac were easily and completely removed manually (Figure 5). After lavage and joint reduction, the head of the rectus femoris was reattached, which was followed by fascia reattachment and skin closure.

**Figure 6:**
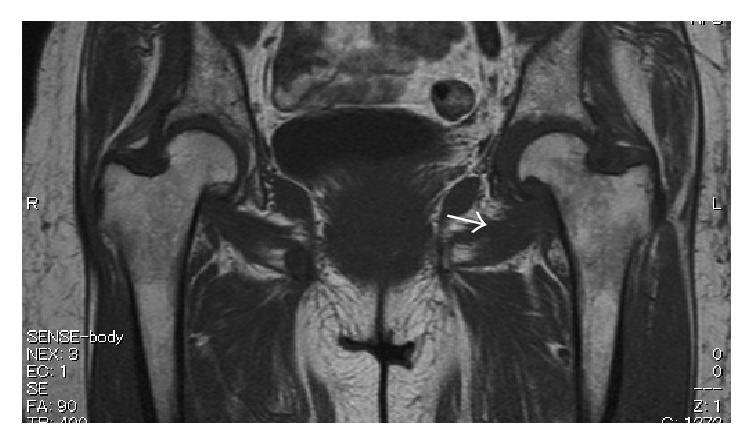
T1-weighted coronal magnetic resonance image at 1 month after surgery. There was no sign of avascular necrosis of the femoral head. The herniation sac had diminished in size (arrow).

The patient began partial weight-bearing gait 2 weeks after surgery and progressed to full weight-bearing at 4 weeks after surgery. MR imaging performed 1 month after surgery revealed no sign of avascular necrosis of the femoral head. The herniation sac diminished in size, with no apparent contents (Figure 6). At follow-up 3 years after surgery, there was no evidence of tumour recurrence or of osteoarthritic changes. The patient was symptom-free, with full range of motion of the left hip. Her Merle d'Aubigné-Postel score had improved (18 points).

Case 2 involved a 45-year-old woman who reported continuous left hip pain since March 2015. She arrived at our hospital in November 2015. She also had a limited range of motion of her left hip. Her Merle d'Aubigné-Postel score was 10 points, with the following distribution: pain, 2 points; walking ability, 4 points; and mobility, 4 points.

**Figure 7:**
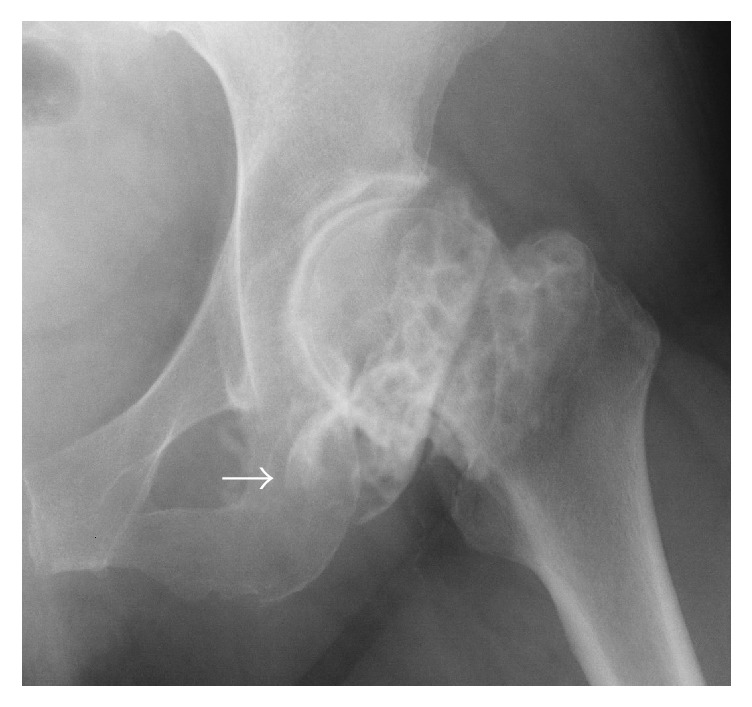
Arthrography of the hip. There are many loose bodies in the left hip joint (honeycomb appearance: arrow).

**Figure 8:**
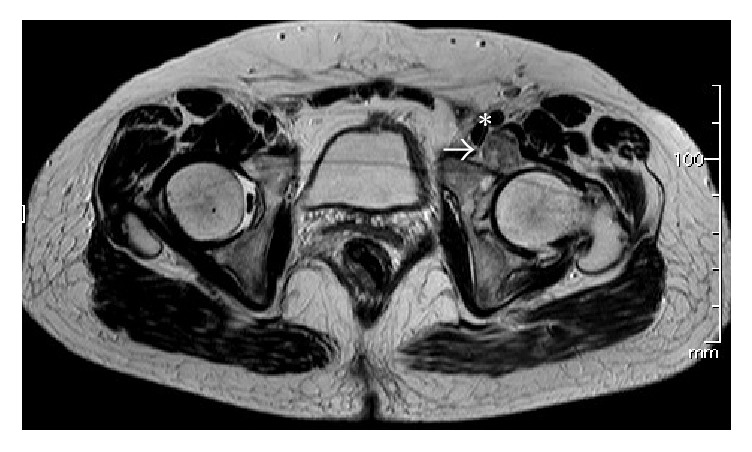
T1-weighted axial image of extra-articular loose bodies (arrow) adjacent to the iliopsoas muscle and the femoral vessels (asterisk).

Arthrography showed a honeycomb appearance around her left hip joint (Figure 7). Upon MR imaging, axial images of the left hip joint revealed intra-articular and extra-articular mass lesions, one of which was located adjacent to the femoral vessels (Figure 8). These examination findings suggested synovial osteochondromatosis forming a herniation sac. Based on the radiographic features, we considered that arthroscopic resection concomitant with an open anterior approach was suitable for complete resection.

**Figure 9:**
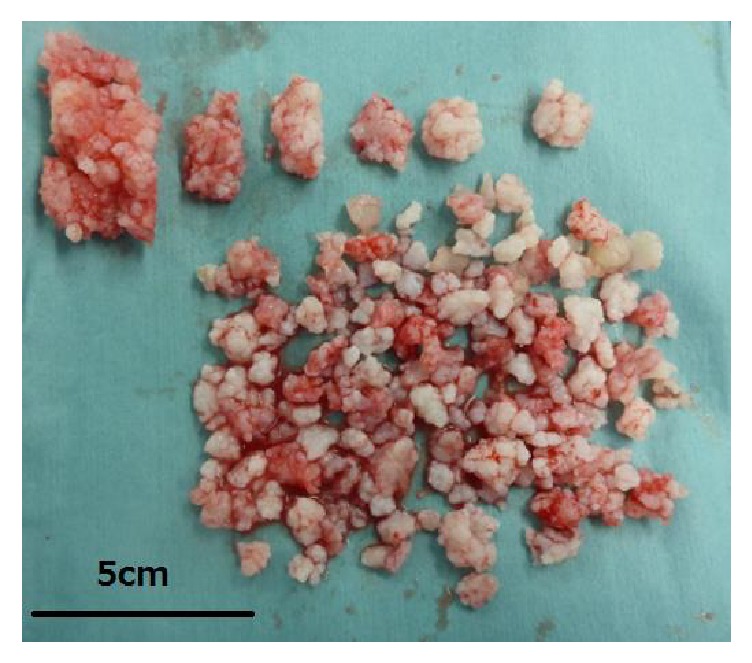
Excised osteochondromas of case 2.

The salient components of the surgical procedure were as follows. The patient was placed in the supine position on the fracture table and arthroscopic resection was performed. We could excise free bodies and osteochondromas existing in the acetabular fossa and the recess behind the labrum, but we could not excise those in the iliopsoas muscle and in the herniation sac near the obturator external muscle bursa. Then, we finished the arthroscopic resection and performed open surgery using an anterior approach. The anterior capsule was opened with a reverse T-shape incision. Osteochondromas remaining in the joint and those in the herniation sac near the obturator external muscle bursa were easily resected without surgical dislocation. Osteochondromas existing in the iliopsoas muscle were completely excised by bluntly splitting the muscle and herniation sac (Figure 9). The capsule was repaired and fascia and skin closure was performed.

The patient began partial weight-bearing gait immediately after surgery and progressed to full weight-bearing at 4 weeks after surgery. Histopathological examination confirmed the diagnosis of a synovial osteochondromatosis. MR imaging performed 1 year after surgery showed that the herniation sac had diminished in size, with no apparent complications. At 1-year follow-up after surgery, the patient had slight pain in her left hip during activity and an improved range of motion. Her Merle d'Aubigné-Postel score improved to 17 points.

## 3. Discussion

A few studies have described successful arthroscopic excision of synovial osteochondromatoses of the hip [2, 3]. Although most of these cases of osteochondromatosis were limited to the joint, the tumour had spread to an extra-articular site in a few cases. Once thought to be a rare occurrence, extra-articular invasion in 21% of synovial osteochondromatoses cases has been reported [1]. For cases of extra-articular invasion with synovial osteochondromatosis, it is difficult and time-consuming to completely excise the tumour using arthroscopy. According to Lee et al. [3], an arthroscopic approach to the posterolateral and posteromedial areas of the peripheral compartment of the hip is technically difficult. In addition, an arthroscopic approach to the peripheral compartment distal to the zona orbicularis increases the risk of injury to the nutrient vessels of the head of the femur, such as a branch of the medial circumflex femoral artery [8, 9]. However, surgical dislocation is often needed for an open approach to completely excise a massive synovial osteochondromatosis.

Ganz et al. [4] provided evidence that the head of the femur can be safely dislocated using an anterior (Smith-Petersen) approach. However, inspection of the acetabulum is limited unless the tensor fascia latae and gluteus medius are extensively detached from their origins. In our case, excision of the osteochondroma from the acetabular fossa was easily completed without damage to the tensor fascia latae and gluteus medius muscles using a curette, with direct inspection and complete excision secured with an arthroscope. Another option would have been to use a lateral approach. However, lateral dislocation of the hip includes the possibility of many complications, including heterotopic ossification, nonunion of the greater trochanter, and nerve traction palsies [4, 10].

Although there is a classic report of the anterior approach used in the supine position for the resection of a synovial osteochondromatosis [11], to our knowledge, the combination of arthroscopic resection and open surgery using an anterior approach with or without surgical hip dislocation has not been previously reported. An anterior approach has become popular for total hip arthroplasty. Surgical dislocation using an anterior approach is less invasive and can safely provide wide exposure of the acetabulum, thereby reducing the risk of damaging the blood supply to the head of the femur. No trochanteric osteotomy is necessary.

When planning our surgical approach, we also considered manual excision to be more desirable because the herniation sac was located adjacent to the obturator foramen, iliopsoas muscle, and surrounding vessels. Using an anterior approach, we could easily reach the herniation sac and completely remove the osteochondromas without complications. The anterior approach with or without surgical dislocation of the hip can be considered an option for excision of a massive synovial osteochondromatosis of the hip joint.
